# A Complementary Herbal Product for Controlling Giardiasis

**DOI:** 10.3390/antibiotics10050477

**Published:** 2021-04-21

**Authors:** Tarek Hamdy Abd-Elhamid, Iman A. M. Abdel-Rahman, Amany Refaat Mahmoud, Khaled S. Allemailem, Ahmad Almatroudi, Samer S. Fouad, Osama H. Abdella, Hatem A. Elshabrawy, Asmaa M. El-Kady

**Affiliations:** 1 Department of Histology and Cell Biology, Faculty of Medicine, Assiut University, Assiut 71515, Egypt; tabdelhamid@aun.edu.eg; 2 Department of Pharmacognosy, Faculty of Pharmacy, South Valley University, Qena 83523, Egypt; emanabdelraheem@svu.edu.eg; 3 Department of Human Anatomy and Embryology, Faculty of Medicine, Assiut University, Assiut 71515, Egypt; armahmoud74@gmail.com; 4 Department of Basic Medical Sciences, Unaizah College of Medicine and Medical Sciences, Qassim University, Unaizah 51911, Saudi Arabia; 5 Department of Medical laboratories, College of Applied Medical Sciences, Qassim University, Buraydah 51452, Saudi Arabia; K.allemailem@qu.edu.sa (K.S.A.); aamtrody@qu.edu.sa (A.A.); 6 Qena University Hospital, South Valley University, Qena 83523, Egypt; samer.saad80@yahoo.com; 7 Department of Medical Parasitology, Faculty of Medicine, South Valley University, Qena 83523, Egypt; osama_hussein1891@med.svu.edu.eg; 8 Department of Molecular and Cellular Biology, College of Osteopathic Medicine, Sam Houston State University, Conroe, TX 77304, USA

**Keywords:** giardiasis, NO, IL-6, TNF-α, IFN-γ

## Abstract

Giardiasis is an intestinal protozoal disease caused by *Giardia lamblia.* The disease became a global health issue due to development of resistance to commonly used drugs. Since many plant-derived products have been used to treat many parasitic infestations, we aimed to assess the therapeutic utility of *Artemisia annua* (*A. annua*) for giardiasis. We showed that NO production was significantly reduced whereas serum levels of IL-6, IFN-γ, and TNF-α were elevated in infected hamsters compared to uninfected ones. Additionally, infection resulted in increased numbers of intraepithelial lymphocytes and reduced villi heights, goblet cell numbers, and muscularis externa thickness. We also showed that inducible NO synthase (iNOS) and caspase-3 were elevated in the intestine of infected animals. However, treatment with *A. annua* significantly reduced the intestinal trophozoite counts and IEL numbers, serum IL-6, IFN-γ, and TNF-α, while increasing NO and restoring villi heights, GC numbers, and ME thickness. Moreover, *A. annua* treatment resulted in lower levels of caspase-3, which indicates a protective effect from apoptotic cell death. Interestingly, *A. annua* therapeutic effects are comparable to metronidazole. In conclusion, our results show that *A. annua* extract is effective in alleviating infection-induced intestinal inflammation and pathological effects, which implies its potential therapeutic utility in controlling giardiasis.

## 1. Introduction 

Giardiasis is one of the most common human intestinal protozoal infections [1]. It is caused by *Giardia lamblia* (*G. lamblia*; also known as *Giardia duodenalis*), which is a flagellated protozoan infecting the small intestine of humans and animals [2]. Approximately 280 million people worldwide are infected with *G. lamblia* annually [3]. The prevalence rates were estimated at 20–30% and 2–5% in developing and developed countries, respectively [4]. In Egypt, the prevalence rate of giardiasis ranges from 10 to 35%, making Egypt an endemic region according to the World Health Organization (WHO) [5,6,7,8].

Giardiasis is transmitted through the ingestion of cysts in food and water. Infection can remain asymptomatic or can present in the form of acute or chronic diarrhea that is accompanied by abdominal pain and nausea [9]. Children can also present with malabsorption and loss of weight, which may impact their cognitive abilities [10,11,12]. Moreover, it has been shown that giardiasis can be more severe in immunocompromised patients [13].

Pathological changes in *G. lamblia*-infected intestines have been described and the organism is usually found on the villus surface or between the intestinal villi [14]. Infection with *G*. *lamblia* is associated with mucosal inflammation, intraepithelial inflammatory cell infiltration, and changes in the villus architecture [15]. Additionally, increased mucus production has been observed in the intestine of an animal model of giardiasis [16]. Moreover, giardiasis induces enterocyte apoptosis which distorts the intestinal mucosal barrier [17,18]. Furthermore, enteric neurons and smooth muscles are affected, which leads to reduced thickness of the external muscle layer of the intestine [19].

Previous studies of giardiasis in rodents have highlighted the essential role of IL-6 in promoting immunity against *G. lamblia* with elevated IL-6 mRNA levels in infected mice [20]. Interestingly, IL-6-deficient mice failed to effectively clear *G. lamblia* infection [20,21]. IgA production in these IL-6-deficient mice was uncompromised, which suggests that IL-6 exerts its anti-giardia effect through IgA-independent mechanism. Recently, dendritic cells have been identified as producers of IL-6 that promote clearance of *G. lamblia* infection in mice [22,23]. Studies have shown that intestinal nitric oxide (NO), produced by nitric oxide synthases (NOSs) using arginine as the substrate, contributes to the clearance of *G. lamblia* trophozoites [24,25]. In fact, NO is an essential molecule in the host defense against many pathogens, including *G. lamblia,* due to its cytotoxicity [24,26]. Interestingly, *G. lamblia* trophozoites compete with the host for arginine [27], or increase the host production of arginase which breaks down arginine [28]. The previous mechanisms diminish NO production, thereby promoting the trophozoites’ survival [29]. Therefore, therapeutics that promote NO production could be effective in controlling giardiasis.

Drugs such as furazolidone, quinacrine, paromomycin, benzimidazole compounds, 5-nitroimidazole compounds, and nitazoxanide have long been used in treatment of giardiasis [30]. However, they have been associated with undesirable effects such as gastrointestinal upset, hepatic and renal toxicity, dermatitis, leukopenia, ototoxicity, severe pancreatitis, and high incidences of congenital anomalies [31,32]. Moreover, several studies have reported resistance of *G. lamblia* to these compounds [33,34]. Consequently, there is an urgent need for the discovery of alternative therapeutics for giardiasis.

Medicinal plants such as *Artemisia* have long been used to treat various human diseases [35,36]. This is attributed to their bioactive natural ingredients and their high safety profile [37,38]. Artemisinin, the main sesquiterpene isolated from *Artemisia annua* (*A. annua*), is highly effective in the treatment of quinine-resistant malaria and is a potent antioxidant due to its high phenolic content [39,40]. Several studies have also shown that *A. annua* is effective against trypanosomiasis [41], schistosomiasis [42], toxoplasmosis [43], leishmaniasis [44,45], and coccidiosis [46]. Moreover, in vitro experiments have demonstrated the anti-giardia effect of *A. annua* [47].

In the current study, we evaluated the efficacy of *A. annua* in an animal model of giardiasis. We show that *A. annua* is effective in reducing *G. lamblia* trophozoite count, inflammation, and parasite-induced intestinal pathological changes. Interestingly, all these effects were comparable to metronidazole, the currently used anti-giardiasis drug, which suggests that *A. annua* could be a potential and safe therapeutic for giardiasis.

## 2. Materials and Methods

This study was conducted at the animal house and Medical Parasitology Department, Faculty of Medicine, South Valley University, Qena, Egypt. All experiments were performed according to the regulations of Animal Care and Use Committee at the Faculty of Medicine, South Valley University, Qena, Egypt. The study design was approved by the Institutional Research Committee at the Faculty of Medicine, South Valley University, Qena, Egypt.

### 2.1. Animals

Thirty-two male golden hamsters, aged 3–4 weeks and weighing 150–200 g each, were procured from the animal house at Theodore Bilharz Research Institute (TBRI), Giza, Egypt. Hamsters were bred under specified pathogen-free conditions. To ensure that hamsters were free from intestinal parasites, stool samples were examined for three consecutive days before starting the experiments

### 2.2. Plant Material

The plant material was collected during the flowering stage from a cultivated field near South Valley University. A voucher specimen of the plant (code: Aa.78) was kept in the herbarium, Department of Pharmacognosy, Faculty of Pharmacy, South Valley University, Egypt. Collected fresh plant was washed with running tap water to remove any associated soil particles. The plant was allowed to dry at room temperature before being ground into a fine powder.

### 2.3. Preparation of A. annua Extract

*A. annua* ethanolic extract was prepared according to the method described previously [48]. *A. annua* ethanolic extract was prepared by maceration of 200 g powdered plant material with 1 L of 95% ethanol. Plant material was allowed to macerate for 16 h at room temperature and then filtered. The process was repeated three times. The combined filtrates were evaporated to dryness in a rotary evaporator under reduced pressure to produce the crude dry ethanolic extract. The obtained dry extract (16 g) was stored at 4 °C for subsequent preparation of the required animal experiment doses.

### 2.4. Preparation of Parasite for Infection

*G. lamblia* cysts were collected from stools of patients of the outpatient clinics in the University hospitals, South Valley University, Qena, Egypt. All stool specimens were processed immediately at the parasitology laboratory. Giardia cyst viability was confirmed using 0.1% eosin vital staining. Cysts were then counted in 0.1 mL of sediment and the concentration process was repeated with more stool samples until the suspension contained about 10,000 viable cysts/mL of phosphate-buffered saline. Animals of different groups were each infected orally with 1 mL of phosphate-buffered saline containing 10,000 cysts [49].

### 2.5. Animal Experiments

Animals were equally divided into 4 groups, 8 animals each. UI group included uninfected animals. Animals of IGL, metronidazole (Met) treatment positive control group, and *A. annua* (AA) group were infected with 1 mL phosphate-buffered saline containing 10,000 giardia cysts orally [49]. Three weeks post-infection, Met and AA animal groups were treated orally with 120 mg/kg of metronidazole for 2 successive days or 400 mg/kg *A. annua* ethanolic extract for 3 consecutive days, respectively [50,51]. Stainless steel esophageal tube was used to administer the treatment. Animals of all groups were then sacrificed 2 weeks after treatment, as previously reported, to evaluate the drug efficacy [50]. Doses of metronidazole and *A. annua* ethanolic extract were selected according to previously published reports [51,52].

### 2.6. Assessment of the Efficacy of the Extract

#### 2.6.1. Determination of Trophozoites Count in Different Groups of Infected Hamsters

After sacrificing hamsters, the small bowel was removed and the duodenal contents were subjected to parasitological examination in order to count the number of *G. lamblia* trophozoites in five successive fields/animal as previously described [53,54]. 

#### 2.6.2. Quantification of Proinflammatory Cytokines and NO 

Blood samples were collected and sera were separated and stored at −20 °C until used. Levels of NO were determined by measuring NO end products (NOx) in serum using Biodiagnostic colorimetric assay kit [55]. The levels of IL-6, TNF-α, and IFN-γ were determined using ELISA kits according to the manufacturer’s protocol (Koma Biotech Inc., Cat. No. K0331229, K0331196, and K0331209, respectively). 

### 2.7. Histopathological Studies

Specimens of 2–5 cm from the proximal part of the small intestine (duodenum and jejunum) removed from sacrificed hamsters were fixed in 10% formalin and embedded in paraffin. Sections of 5 µm thickness were stained with hematoxylin and eosin or Periodic Acid Schiff (PAS) and hematoxylin as previously described [56].

#### 2.7.1. Assessment of Villi Length and Muscularis Externa Thickness

Three animals/group were used for the assessment of these parameters. A total of 15 images of random fields/group were used. Images at 100× magnification were used for assessment of the villi length and at 400× magnification for measuring muscularis externa thickness using Fiji ImageJ software, version 1.52p.

#### 2.7.2. Determination of Intraepithelial Lymphocytes (IEL) and Goblet Cell Numbers

Three animals/group were used for the assessment of IEL and goblet cell numbers. Images captured at 200× were used for assessing IEL. We counted 2500 enterocytes and IELs and then the ratios of IEL/100 enterocytes were calculated [57]. For goblet cell counts, fifteen images of random fields/animal group of sections stained with Periodic Acid Schiff (PAS) and hematoxylin were used. In each image, we counted the number of goblet cells and enterocytes and then we calculated the ratios of goblet cells/100 enterocytes

#### 2.7.3. Immunohistochemistry

Paraffin sections of the small intestine of different groups were deparaffinized and rehydrated with descending percentages of ethanol. Sections were boiled in citrate buffer (pH 6.0) in a microwave for epitope retrieval. Endogenous peroxidases were then blocked with 3% H_2_O_2_ in ethanol. Sections were incubated with anti-caspase-3 antibodies (Thermo Scientific, Waltham, MA, USA; dilution 1:500) or anti-inducible nitric oxide synthase (iNOS) polyclonal antibodies (Thermo Scientific, USA, dilution 1:50) for 60 min at room temperature. Next, sections were washed with TBS containing 0.05% Tween-20 (TBS-T). Sections were then incubated with HRP-conjugated goat anti-rabbit secondary antibodies (Vivantis Technologies, Malaysia) at a dilution of 1:5000 for 1 h at 4 °C. After washing in TBS-T, the color was developed by incubating sections with 0.05% diaminobenzidine (DAB) and 0.01% H_2_O_2_ for 3 min. For negative control, we omitted the primary antibodies during staining of some slides. Images were captured using Leica light microscope equipped with a digital camera in the Histology and Cell Biology Department, Assiut University, Faculty of Medicine, Assiut, Egypt.

### 2.8. Statistical Analysis

The collected data were analyzed by SPSS (Statistical Package for Social Sciences), version 20 for Windows. All values were expressed as mean ± standard deviation (SD). Differences between groups were determined using one-way ANOVA test to compare the mean values between treated and control groups for different variables by the Bonferroni post hoc test. Differences were considered significant when *p* < 0.05.

## 3. Results

### 3.1. A. annua Treatment Reduces G. lamblia Trophozoite Count in the Intestine

**Figure 1:**
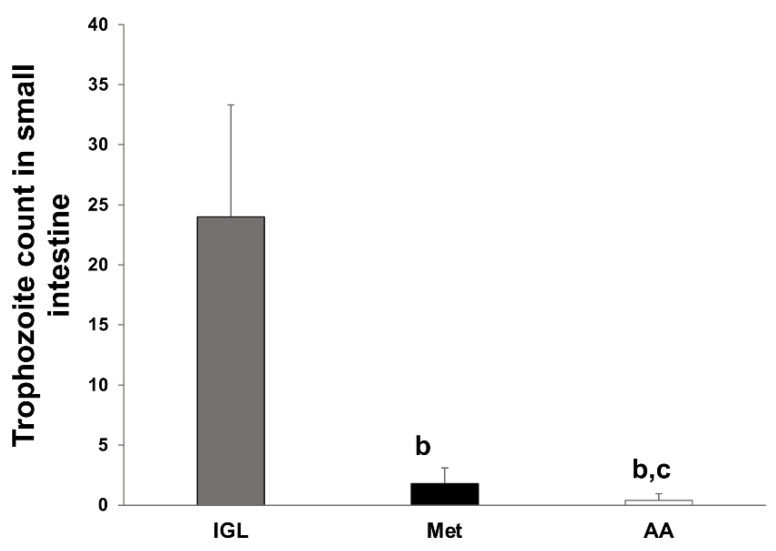
**Treatment with *A. annua* reduced trophozoite counts in the small intestine of infected hamsters.** Trophozoites were counted in infected untreated hamsters (IGL) and compared to counts in animals treated with metronidazole (Met) or *A. annua* (AA) extract. Data are expressed as mean ± SD (n = 8) and were analyzed using ANOVA with Bonferroni corrections for pairwise comparison. Letter “**b**” indicates significant reductions in trophozoite counts after treatments compared to IGL group (*p* < 0.001). Letter “**c**” indicates a significant difference in trophozoite count between Met and AA group (*p* < 0.001).

Examination of small intestines of metronidazole (Met) and *A. annua* (AA) treatment groups for trophozoite count revealed that these animals had significant reductions in trophozoite count compared to the infected untreated group (IGL) (98.3% and 92.5% reductions respectively) (Figure 1). These data suggest that *A. annua* extract is effective in reducing *G. lamblia* trophozoite count in the small intestine.

### 3.2. A. annua Treatment Induced NO and Reduced Proinflammatory Cytokines Production

**Figure 2:**
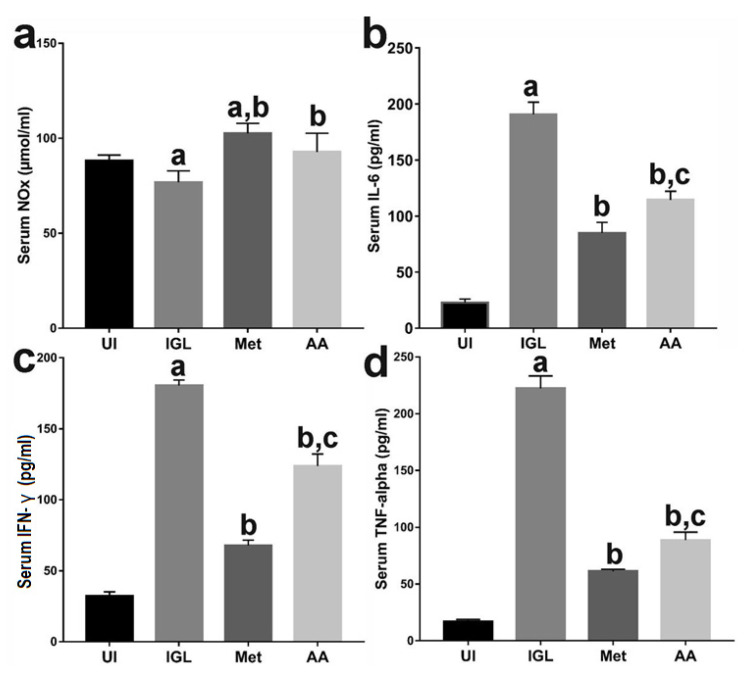
**The effect of *A. annua* treatment on NO and proinflammatory cytokine levels in hamsters infected with *G. lamblia***. We measured NO end products (NOx) in serum of different groups (**a**) (“a” indicates a significant difference versus UI group (*p* < 0.001), while “b” indicates a significant difference versus IGL (*p* < 0.001)). Serum levels of IL-6 (**b**), IFN-γ (**c**), and TNF-α (**d**) were also quantified (“a” indicates a significant difference versus UI group, “b” indicates a significant difference versus IGL group, and “c” indicates a significant difference versus Met group (*p* < 0.05)). Values represent means ± SD (n = 8), and data were analyzed using ANOVA with Bonferroni corrections as a post hoc test. UI = uninfected animal group, while IGL = infected and untreated animals. Met = infected animals treated with metronidazole and AA = infected animals treated with *A. annua* extract.

The role of NO in eradicating *G. lamblia* infection has been well established [24]. Therefore, we sought to quantify NO production in different animal groups by measuring NO end products (NOx) in serum. We found that infection with *G. lamblia* (IGL group) significantly reduced NO compared to uninfected animals (UI). In contrast, treatment of infected hamsters with metronidazole (Met group) significantly increased NO compared to IGL group (*p* < 0.001) and UI group (*p* < 0.001). Similarly, treatment with *A. annua* extract (AA group) significantly induced NO production compared to IGL group (*p* < 0.001). However, there was no significant difference between metronidazole and *A. annua* treatment on NO production (Figure 2a).

Next, we measured serum IL-6 levels in different animal groups. Zhou et al. previously reported increased IL-6 levels during the course of giardiasis in mice [20]. Therefore, we sought to compare the effects of metronidazole and *A. annua* treatments on giardiasis-induced increase in IL-6 serum levels. IL-6 was significantly higher in sera of animals infected with *G. lamblia* (IGL group) compared to uninfected animals (UI group) (*p* < 0.05). Treatment of infected animals with metronidazole (Met group) or *A. annua* extract (AA group) resulted in a significant reduction in serum IL-6 compared to IGL group (*p* < 0.05) (Figure 2b). However, metronidazole significantly reduced IL-6 when compared to *A. annua* treatment (*p* < 0.05) (Figure 2b). A recent study by Pacheco et al. has demonstrated elevated IFN-γ in children with giardiasis [58]. Therefore, we sought to compare the effects of metronidazole and *A. annua* treatments on giardiasis-induced IFN-γ. As expected, animals infected with *G. lamblia* (IGL group) showed significant increase in the serum IFN-γ compared to the uninfected animals (UI group) (*p* < 0.05) (Figure 2c). On the other hand, the serum IFN-γ levels were significantly lower in hamsters treated with metronidazole or *A. annua* compared to IGL group (*p* < 0.05) (Figure 2c). However, similar to IL-6, serum IFN-γ levels were significantly lower in the Met group than AA group (Figure 2c). We also investigated the serum TNF-α levels in infected animals treated with metronidazole or *A. annua* extract. As shown in Figure 2d, serum levels of TNF-α in IGL group were significantly elevated compared to the UI animals (*p* < 0.05). However, treating infected animals with metronidazole or *A. annua* extract significantly reduced serum TNF-α levels compared to the infected untreated hamsters by 4-fold and 3-fold, respectively. 

### 3.3. A. annua Treatment Restored Villi Structure of G. lamblia-Infected Animals

**Figure 3:**
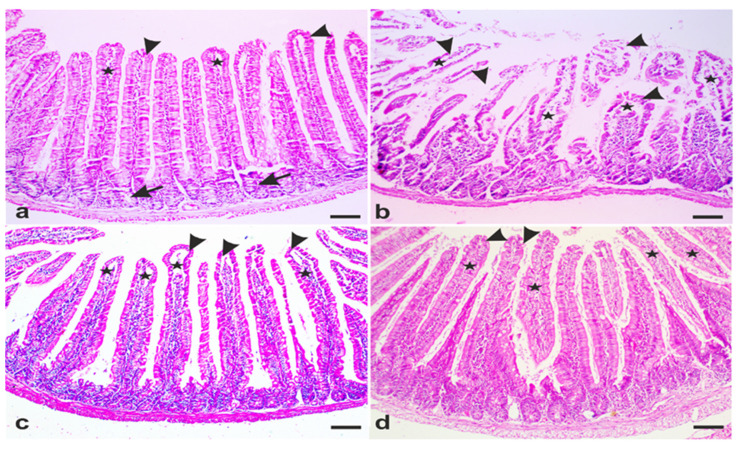
**Representative photomicrographs of small intestinal sections from different groups of hamsters stained with hematoxylin and eosin.** (**a**) Representative tissue from uninfected animals showing intact villi (arrowheads), crypts (arrows), connective core of the villi (stars). (**b**) Representative tissue from infected animals showing shortening and disruption of villi (arrowheads) and retraction of the connective tissue core of the villi (stars). (**c**) Representative tissue from infected animals that are treated with metronidazole with well-formed mucosal epithelial lining of the villi (arrowheads) and intact villi cores (stars). (**d**) Representative tissue from infected animals that are treated with *A. annua* extract showing well-formed villi (arrowheads) with regular epithelial lining and intact connective tissue core (stars). Scale bars = 100 µm.

To further explore the effects of metronidazole and *A. annua* treatments on giardiasis-induced small intestinal lesions, we stained sections taken from the proximal parts of the small intestine with hematoxylin and eosin (H&E) stains. Small intestinal sections taken from UI animals showed normal intestinal villi and crypts (Figure 3a, arrowheads and arrows). The core of the villi is formed of connective tissue (Figure 3a, stars). In contrast, marked shortening and destruction of intestinal villi together with retraction of their connective tissue cores were observed in small intestinal sections taken from infected untreated animals (Figure 3b, arrowheads and stars). On the other hand, treating infected animals with metronidazole or *A. annua* restored the structure of the villi and their connective tissue cores (Figure 3c,d; arrowheads and stars).

**Figure 4:**
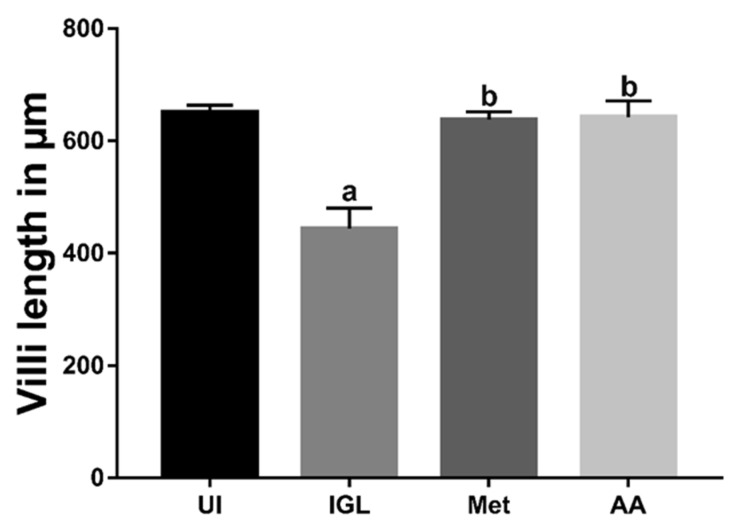
**Treatment with*A. annua* preserved villi length in infected hamsters.***A. annua* extract treatment protected villi in infected hamsters. This effect was comparable to metronidazole treatment. “**a**” indicates significant difference versus UI group and “**b**” indicates significant difference versus IGL group (*p* < 0.001). Data are presented as means ± SD (n = 3) and were analyzed using ANOVA test to compare the mean differences between groups with Bonferroni corrections as a post hoc test.

Next, we measured the villi length and found a significant reduction in the villi length in sections taken from IGL group animals compared to UI animals (*p* < 0.001) (Figure 4). However, treating infected animals with metronidazole (Met) or *A. annua* (AA) restored the normal villi length (*p* < 0.001). We did not find a significant difference between the villi length in Met and AA group.

**Figure 5:**
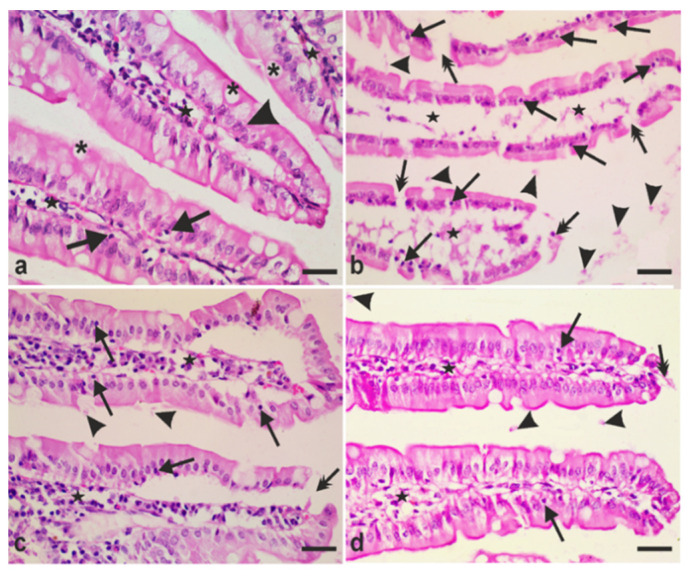
**Representative photomicrographs of small intestinal tissues of different groups of hamsters stained with hematoxylin and eosin**. (**a**) Representative tissue from uninfected animals showing intact intestinal villi that are covered with simple columnar epithelium (arrowheads) with goblet cells (asterisks), villi core (stars), and a few IELs (arrows). (**b**) Representative tissue from infected untreated animals showing disruption of the mucosal epithelium (double-headed arrows), marked increase in IELs (arrows), retracted villi core with a few scattered connective tissue cells (stars), and presence of giardia trophozoites between villi (arrowheads). (**c**) Representative tissue from infected animals treated with metronidazole showing mucosal epithelium with a few desquamated cells (double-headed arrows), regeneration of the villi core (stars), a few IELs (arrows), and giardia trophozoites in the intervillous spaces (arrowheads). (**d**) Representative tissue from infected animals treated with *A. annua* showing preserved intestinal villi with a few desquamated cells (double-headed arrows) and intraepithelial lymphocytes (arrows). Stars point to intact villi core, whereas arrowheads point to a few giardia trophozoites found between the villi. Scale bar = 30 µm.

At higher magnification, small intestinal sections taken from uninfected hamsters exhibited normal histological structure of the villi with surface simple columnar epithelial cells (Figure 5a, arrowheads) and goblet cells (Figure 5a, asterisks). A few intraepithelial lymphocytes (IELs) were also observed (Figure 5a, arrows) and the core of the villi is formed of connective tissue (Figure 5a, stars). On the other hand, sections from infected animals showed desquamation of epithelial cells, disruption of the epithelium covering the villi (Figure 5b, double-headed arrows), increased IELs (Figure 5b, arrows), and disintegration of the connective tissue core of the villi with a few scattered connective tissue cells in this core (Figure 5b, stars). We also observed *G. lamblia* trophozoites in the intervillous spaces (Figure 5b, arrowheads). However, tissue sections of animals treated with metronidazole showed normal villi epithelium except for a few areas of epithelial disruptions (Figure 5c, double-headed arrow), regeneration of the connective tissue core of the villi (Figure 5c, stars), and decreased IEL infiltration (Figure 5c, arrows). Additionally, a few trophozoites were observed in the intervillous spaces (Figure 5c, arrowheads). Similar to metronidazole-treated group, *A. annua* treatment protected epithelial cells covering the villi, with only a few desquamated cells (Figure 5d, double-headed arrows), a few IELs (Figure 5d, arrows), and well-preserved villous connective tissue core (Figure 5d, stars). A few trophozoites were seen in the intervillous regions (Figure 5d, arrowheads).

### 3.4. A. annua Treatment Reduced IEL Numbers in G. lamblia-Infected Animals, Restored the Goblet Cell Numbers, and Reversed Infection-Induced Pathological Effect on Muscularis Externa

**Figure 6:**
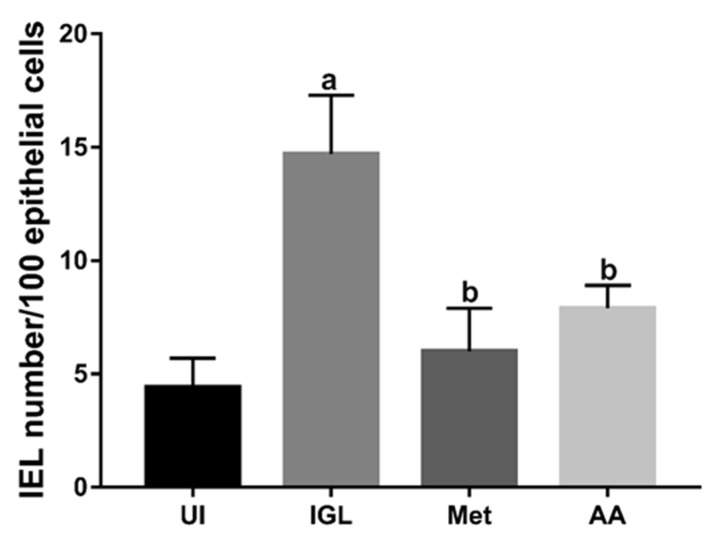
**Treatment with*A. annua* reduced the number of intestinal IELs in infected hamsters.** IELs were counted in images of intestinal tissues stained with hematoxylin and eosin. Infection of hamsters with *G. lamblia* significantly increased IEL count compared to uninfected animals (IGL versus UI). Treatment with *A. annua* significantly decreased IEL count compared to infected untreated animals (AA versus IGL). No significant difference was found between metronidazole or *A. annua* treatment (Met versus AA). “**a**” indicates significant difference versus UI group and “**b**” indicates significant difference versus IGL group (*p* < 0.001). Data are expressed as means ± SD (n = 3) and were analyzed using ANOVA test with Bonferroni corrections as a post hoc test.

Next, we counted the IELs/100 epithelial cells on images of H&E-stained sections taken at 200× magnification according to previously published methods [57]. As shown in Figure 6, infected hamsters (IGL) had a significant increase in IEL numbers compared to uninfected (UI) animals (*p* < 0.001). However, treatment with metronidazole (Met) or *A. annua* (AA) significantly decreased the number of IELs in epithelial cells (*p* < 0.001). We did not find a significant difference between the effect of metronidazole and *A. annua* on the number of IELs.

**Figure 7:**
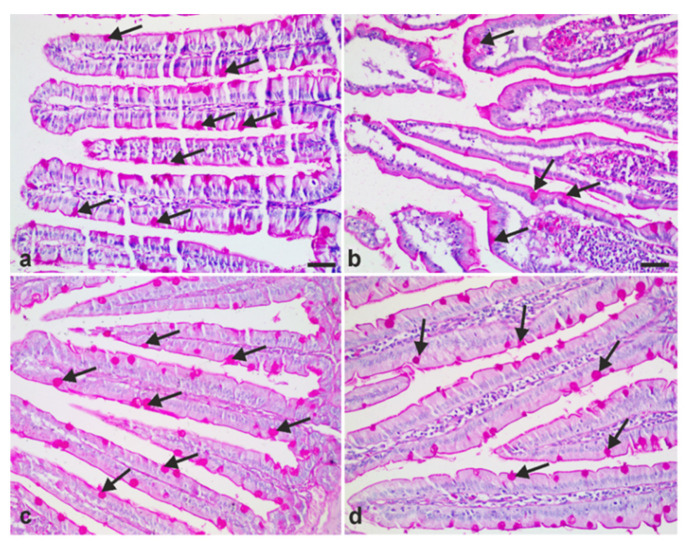
**Representative photomicrographs of small intestinal tissues of different groups of hamsters stained with Periodic Acid Schiff (PAS) and hematoxylin to demonstrate goblet cells.** (**a**) Representative tissue from uninfected animals showing intestinal villi with goblet cells scattered between epithelia cells. (**b**) Representative tissue from infected untreated animals showing depletion of goblet cells. (**c**) Representative tissue from infected animals treated with metronidazole showing restored goblet cells. (**d**) Representative tissue from infected animals treated with *A. annua* with restored goblet cells. Scale bar = 50 µm.

To examine whether *A. annua* treatment can restore goblet cell numbers, we stained our small intestinal sections with Periodic Acid Schiff reagent (PAS) and hematoxylin. In uninfected animals, goblet cells are distributed among the epithelial cells in the epithelium covering the villi (Figure 7a). A few goblet cells were observed in sections taken from infected untreated animals (Figure 7b). However, treating infected animals with metronidazole or *A. annua* reversed *G. lamblia*-induced reduction of goblet cells (Figure 7c,d).

**Figure 8:**
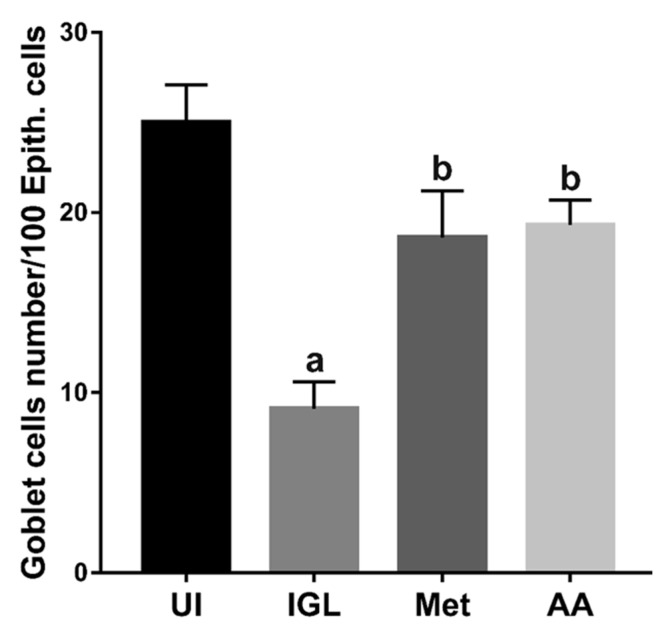
**Treatment with*A. annua* restored goblet cell numbers in small intestine of infected hamsters**. Images of intestinal tissues stained with PAS and hematoxylin were used to count goblet cells. *A. annua* extract significantly increased the goblet cell number compared to infected untreated animals (AA versus IGL). *A. annua* effect was comparable to that of metronidazole. “**a**” indicates significant difference versus UI group and “**b**” indicates significant difference versus IGL group (*p* < 0.001). Data are means ± SD (n = 3) and were analyzed using ANOVA test with Bonferroni corrections as a post hoc test.

To confirm our observations, we counted the number of goblet cells/100 epithelial cells in images of small intestinal sections stained with PAS and hematoxylin. A total of 2500 epithelial cells were counted according to previously published methods [59]. Infection of hamsters with *Giardia lamblia* (IGL group) significantly reduced goblet cell number compared to the uninfected (UI) animals (Figure 8) (*p* < 0.001). On the other hand, treating infected animals with metronidazole (Met) or *A. annua* (AA) significantly increased the goblet cell number compared to the infected untreated animals (*p* < 0.001). No significant difference was found between the effect of metronidazole and *A. annua* on goblet cell number.

**Figure 9:**
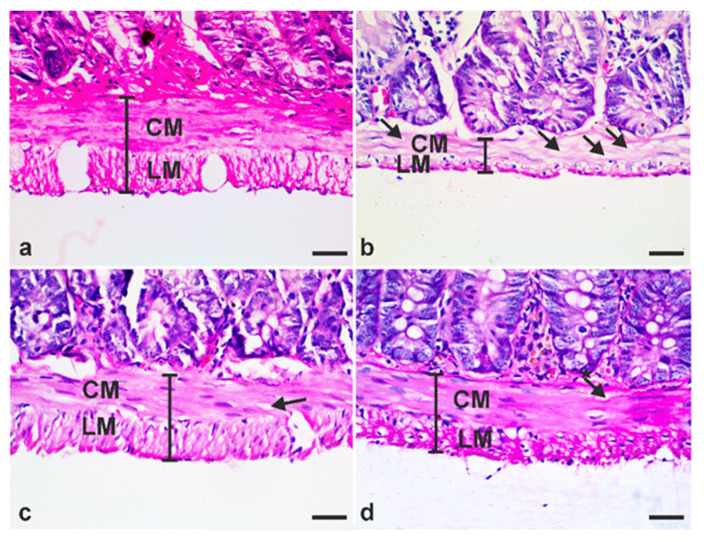
**Treatment with*A. annua* restored pathological changes in muscularis externa due to *G. lamblia* infection.** (**a**) Representative intestinal tissue from uninfected animals showing well-formed muscularis externa composed of smooth muscle fibers arranged in two layers; inner circular (CM) and outer longitudinal (LM) layers. (**b**) Representative intestinal tissue from infected untreated animals with extensively vacuolated muscle fibers (arrows) and decreased thickness of muscularis externa. (**c**) Representative intestinal tissue from infected animals treated with metronidazole with a few muscle fibers with vacuolation (arrows). (**d**) A representative tissue from small intestine of animals infected and treated with *A. annua* extract showing well-formed muscularis externa layers and a few fibers with vacuolation (arrows). In all panels, the vertical lines with brackets indicate the muscularis externa thickness. Scale bar = 30 µm.

Earlier, Pavanelli et al. have demonstrated that giardiasis reduced the muscularis externa thickness [19]. Therefore, we sought to investigate whether treatment of infected animals with either metronidazole or *A. annua* would reverse giardiasis-induced muscularis externa changes. In sections taken from the small intestine of uninfected hamsters, the muscularis externa is composed of smooth muscle fibers arranged in two layers, inner circular and outer longitudinal layers. These cells have acidophilic cytoplasm and elongated vesicular centrally located nuclei (Figure 9a). As expected, small intestinal sections taken from infected untreated hamsters showed extensive vacuolation of muscle cells of the muscularis externa (Figure 9b, arrows). Additionally, there was a reduction in the thickness of the muscularis externa of these animals. In contrast, small intestinal section of animals treated with metronidazole or *A. annua* showed decreased cytoplasmic vacuolation of smooth muscle cells (Figure 9c,d; arrows). Moreover, muscularis externa layers appeared thicker than those of infected untreated animals (Figure 9c,d).

**Figure 10:**
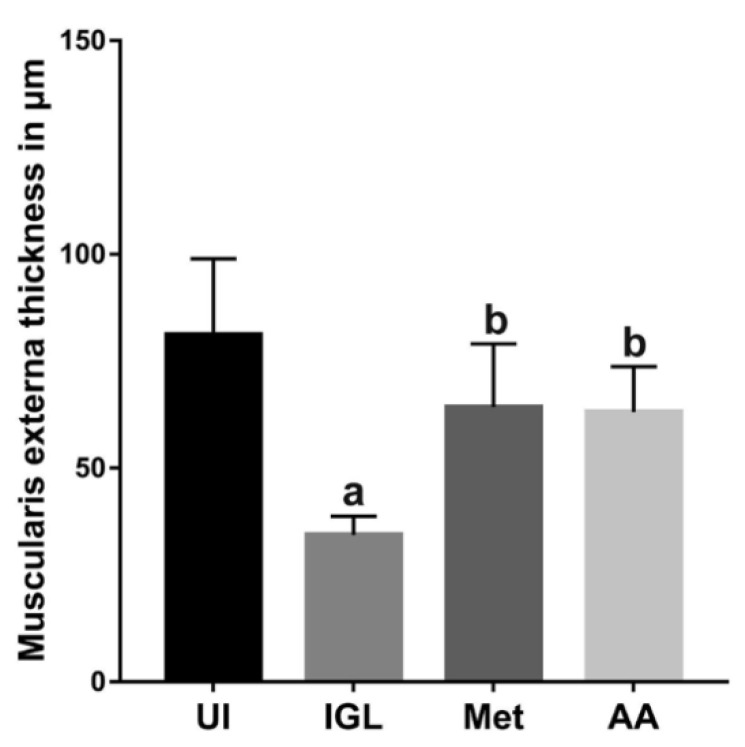
**Treatment with*A. annua* reversed the pathological effect of *G. lamblia* infection on muscularis externa thickness.** Infection of hamsters with *G. lamblia* (IGL) significantly decreased the muscularis externa thickness compared to uninfected animals (UI). Treatment of infected hamsters with *A. annua* extract (AA) significantly increased the muscularis externa thickness compared to infected untreated animals, an effect which was comparable to that after metronidazole treatment (Met). (**a**) indicates significant difference versus UI group. (**b**) indicates significant difference versus IGL group (*p* < 0.001). Data are expressed as means ± SD (n = 3) and were analyzed using ANOVA test with Bonferroni corrections as a post hoc test.

To confirm our results, we measured the thickness of the muscularis externa, using Fiji ImageJ software version 1.52p, in 15 pictures of random fields taken at 400 × magnification. As shown in Figure 10, infection of hamsters with *G. lamblia* significantly reduced muscularis externa thickness compared to the uninfected animals (*p* < 0.001). On the other hand, treating infected animals with metronidazole (Met) or *A. annua* (AA) extract significantly increased the muscularis externa thickness compared to the infected untreated animals (*p* < 0.001). No significant difference was observed between the effects of the two treatments.

### 3.5. A. annua Treatment Modulates iNOS Expression and Protects Intestinal Cells from Apoptosis

**Figure 11:**
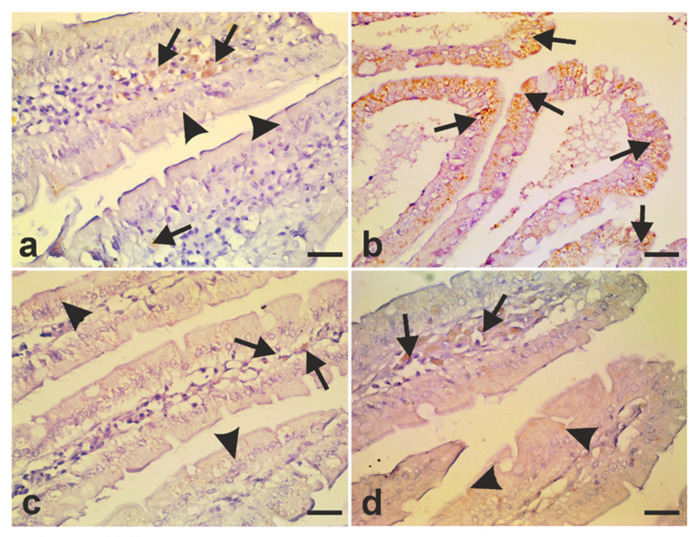
***A. annua* treatment of infected hamsters modulatediNOS expression in small intestine.** (**a**) iNOS expression was localized in core intestinal cells of infected untreated animals (arrows) with no iNOS immunoreactivity in enterocytes. (**b**) Enterocytes showed high expression of iNOS in infected untreated animals (arrowheads). (**c**) Moderate iNOS expression was detected in core cells (arrows) as well as enterocytes (arrowheads) of intestines taken from infected animals that were treated with metronidazole. (**d**) Low iNOS expression was detected in villi core mononuclear cells of infected animals that were treated with *A. annua* extract (arrows) and was barely detected in enterocytes (arrowheads). Scale bar = 30 µm.

A large body of evidence has shown that inducible nitric oxide synthase (iNOS) is highly expressed following *G. lamblia* infection and that NO is important for elimination of the parasite [29,60]. To investigate whether treating infected hamsters with metronidazole or *A. annua* would modulate the expression of iNOS, we employed immunohistochemistry to examine iNOS expression in tissue from the small intestine. In uninfected animals, we showed that iNOS is expressed in connective tissue cells in the core of the villi (Figure 11a, arrows) and barely in the enterocytes (Figure 11a, arrowheads). Infection of hamsters with *G. lamblia* induced intense iNOS signals in enterocytes covering the villi (Figure 11b, arrows). In infected animals that are treated with metronidazole, moderate iNOS expression was observed in villicore connective tissue cells (Figure 11c, arrows) and enterocytes (Figure 11c, arrowheads). In contrast, small intestinal tissues from *A. annua*-treated animals showed a few cells of the villi core expressing iNOS (Figure 11d, arrows) and weak iNOS signals in enterocytes (Figure 11d, arrowheads).

**Figure 12:**
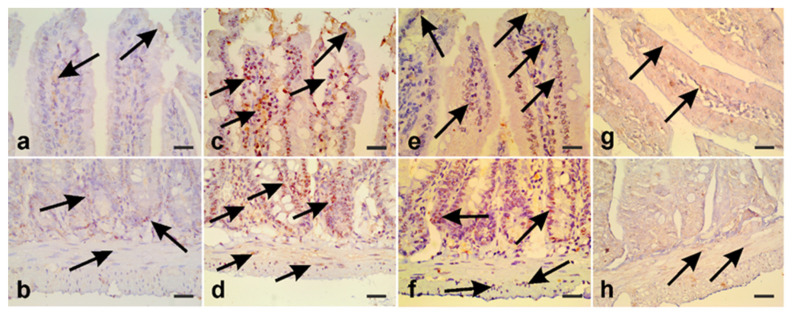
**Treatment with *A. annua* inhibited apoptosis of intestinal cells that is induced by *G. lamblia* infection.** (**a**,**b**) Representative intestinal tissue sections of small intestine, from uninfected hamsters, showing weak caspase-3 signals (arrows). (**c**) Representative intestinal tissue, from infected animals, showing strong caspase-3 signals in enterocytes and lamina propria cells (arrows) and in (**d**) crypts and muscularis externa (arrows). Representative intestinal tissue, from infected animals treated with metronidazole, showing lower levels of caspase-3 in the enterocytes and villi core cells (**e**) (arrows) and in crypt cells and muscularis externa (**f**). (**g**,**h**) showing a very low level of caspase-3 in intestinal tissue of infected animals that are treated with *A. annua*. Scale bar = 30 µm.

Previously, it has been demonstrated that *G. lamblia* trophozoites induce apoptosis in cultured enterocytes [18]. Therefore, we sought to test whether treating infected hamsters with *A. annua* extract would attenuate giardia-induced apoptosis in small intestine. We immunostained our small intestinal sections with anti-caspase-3 antibodies. In uninfected control animals, weak caspase-3 signals were observed in the enterocytes, lamina propria cells (Figure 12a, arrows), crypt cells, and muscularis externa layers (Figure 12b, arrows). In contrast, high levels of caspase-3 were detected in small intestinal epithelial cells and lamina propria cells (Figure 12c, arrows) of infected untreated animals. We also observed strong caspase-3 signals in cells lining the crypts as well as smooth muscle cells of muscularis externa layers (Figure 12d, arrows). Metronidazole-treated animals had fewer caspase-3-positive villi epithelial cells, crypt cells, and muscularis externa cells (Figure 12e,f; arrows). Interestingly, caspase-3 was significantly lower in intestine of *A. annua*-treated animals compared to infected untreated animals and metronidazole-treated animals (Figure 12g,h; arrows). The previous results show that *A. annua* treatment modulates iNOS expression and protects intestinal tissue from apoptotic cell death due to infection with *G. lamblia*.

## 4. Discussion

Giardiasis is a major worldwide health problem that is caused by *G. lamblia* parasite. Studies have reported resistance of this parasite to commonly used drugs [33,34]. However, many research groups have identified the use of plants and plant-derived products, including *A. annua*, for the treatment of parasitic diseases [61,62,63]. Accordingly, in the current study, we used a hamster model of giardiasis to assess the efficacy of ethanolic extract of *A. annua* in *giardiasis*. We show for the first time that *A. annua* ethanolic extract significantly reduces *G. lamblia* trophozoite counts in small intestine of hamsters. Additionally, we show that *A. annua* restored *G. lamblia*-induced reduction of nitric oxide and significantly lowered IL-6, IFN-γ, and TNF-α production due to *G. lamblia* infection. Furthermore, *A. annua* extract reversed *G. lamblia*-induced pathological changes in villi heights, IEL numbers, goblet cell numbers, and muscularis externa thickness. Our results suggest an anti-giardia therapeutic effect of *A. annua* ethanolic extract.

In the present study, *G. lamblia*-infected hamsters treated with *A. annua* extract showed a marked reduction in the number of trophozoites in intestinal tissues in comparison with the infected untreated animals. This finding is in line with Alin and Bjorkman’s study which showed that artemisinin, an *A. annua* active ingredient, inhibited the growth of malaria parasite in a concentration-dependent manner [64]. Additionally, in vitro studies demonstrated that *A. annua* extract killed giardia trophozoites and this effect was concentration- and time-dependent [47]. We believe that reduction of *G. lamblia* trophozoite counts in our study after *A. annua* treatment could be through direct killing or growth inhibition of the trophozoites.

Several studies have suggested that NO plays an important role in the elimination of *G. lamblia* trophozoites [24,65]. To survive in the intestine, *G. lamblia* trophozoites limit host production of NO by competing with the host for arginine, as they use it as an energy source during various stages of growth [27]. Additionally, in vitro experiments have demonstrated that *G. lamblia* trophozoites secrete arginase, which breaks down arginine [66]. Furthermore, Maloney et al. reported an increased production of arginases by small intestinal macrophages during giardiasis [28]. In line with these data, we have shown reduction in serum NO end products (NOx) levels in animals infected with *G. lamblia*. Although we, in the current work, and others have reported increased expression of iNOS in the intestine of infected animals, this failed to increase NO levels due to arginine deficiency [29,60]. However, we have shown that treatment with *A. annua* extract increased NO production (as evidenced by increased serum NOx). This could be attributed to either the inhibitory effect of *A. annua* on *G. lamblia* trophozoite growth or inhibition of arginase release by macrophages. This assumption is partially supported by the work of Yang and colleagues who showed that *A. annua* derivatives inhibited activation of macrophages in vitro [67]. However, the increased NO levels in our study, after *A. annua* treatment, were associated with decreased expression of iNOS. The increased NO production in *A. annua*-treated animals could be explained by Li etal.’s study. This study demonstrated that neuronal nitric oxide synthase (nNOS) plays a more significant role in giardiasis than iNOS [68]. Therefore, *A. annua* could increase NO levels through increased expression of nNOS rather than iNOS. However, this speculation needs further investigation.

Our results also showed significant increase in proinflammatory cytokines, namely; IL-6, TNF-α, and IFN-γ in animals infected with *G. lamblia*. Our findings are in line with previous reports that demonstrated increased cytokine production in TLR2-deficient mice which led to reduction in parasite burden and alleviated giardiasis [69,70,71]. Additionally, Bienz et al. showed that IL-6 deficient mice failed to control *G. lamblia* infection [21]. Moreover, mice deficient in TNF-α have an increased parasite load and prolonged *G. lamblia* infection [69]. However, increased production of inflammatory cytokines during *G. lamblia* infection was associated with pathological changes in the small intestine [72]. In our study, treatment with *A. annua* reduced serum IL-6 levels, TNF-α, and IFN-γ compared to those of infected untreated animals. However, these levels were higher than those of metronidazole-treated animals. Therefore, we believe that treatment with *A. annua* would be advantageous over metronidazole since IL-6 blocks epithelial cells apoptosis and helps in epithelial repair [73,74].

Our histological analyses show that infection of hamsters with *G*. *lamblia* led to shortening of the intestinal villi, shedding, and desquamation of enterocytes. These effects could be due to the secreted parasite proteins with proteolytic activities. These proteins could lead to disruption of cell–cell junctions and enterocyte damage in vitro [75]. Additionally, Scott and colleagues have demonstrated that intraepithelial CD8^+^ T cells destroyed the apical membrane of enterocytes, with loss of brush border and reduced disaccharidases activities [76]. Other experiments have reported that treating small intestinal epithelial cells with IFN-γ disrupts mucosal barrier function through loss of zonula occludens (ZO)-1 and increased epithelial permeability [77]. Moreover, Troeger et al. as well as Fisher et al. have associated enterocyte damage with giardia-induced apoptosis [78,79]. Furthermore, several authors have suggested that TNF-α signaling through TNF-α receptor 1 (TNFR1) induced enterocytes apoptosis in a caspase-dependent manner [80,81,82]. In line with this hypothesis, our immunohistochemical data show that *G. lamblia* infection increased caspase-3 levels, which could account for tissue damage seen in our model. Taken together, small intestinal damage observed in our model could be due to the parasite secretory products and/or because of elevated cytokine levels.

However, treating infected animals with *A. annua* extract markedly decreased desquamated enterocytes with nearly normal villi covering. This effect could be attributable to the direct toxic effect of *A. annua* on *G. lamblia* which could protect enterocytes from the proteolytic enzymes secreted by the parasite. This assumption is partially supported by the work of Jiao and colleagues who demonstrated that *A. annua* leaf extract killed *Eimeria tenella*, an intracellular parasite that infects chicken enterocytes [46]. In the same study, the authors also demonstrated that *A. annua* decreased the expression of the inflammatory markers; nuclear factor-kappa B(NF-kB) and IL-17. Moreover, Sun et al. showed that artesunate, an *A. annua* ingredient, induced apoptosis of lamina propria macrophages and dendritic cells and decreased mucosal expression of TNF-α in a rodent model of colitis [83]. Numerous studies have associated NF-kB and TNF-α with various inflammatory conditions [84,85]. Therefore, controlling the activation of NF-kB and expression of TNF-α could account for the curative effect of *A. annua*. Treating infected animals with *A. annua* extract also decreased serum IFN-γ levels. This effect of *A. annua* could account for alleviation of morphological changes that occurs during *G. lamblia* infection. On the other hand, Jiao et al. have demonstrated that *A. annua* leaves induced apoptosis of *Eimeria tenella*-infected enterocytes [46]. Lang and colleagues have also reported that *A. annua* inhibited the viability of resistant cancer cells [86]. In the same context, our immunohistochemistry data revealed that treating infected animals with *A. annua* extract reduced the amount of caspase-3 in enterocytes. In line with this finding, Yuan et al. demonstrated that *Artemisia* derivatives protected mice liver against the injurious effect of acetaminophen through decreased cellular levels of caspase-3 and -8 in addition to lower serum TNF-α levels [87].Therefore, *A. annua* components could inhibit growth or induce apoptosis of infected/stressed cells as suggested by Deng et al. and Jiao et al. reports [46,88], whereas healthy cells could be protected by *A. annua* derivatives as shown by us and others [87]. Altogether, *A. annua* attenuated giardia-induced enterocytes damage through direct toxic effect on *G*. *lamblia*, anti-inflammatory and possible anti-apoptotic effect. However, these mechanisms of *A. annua* therapeutic effect need further investigation.

In the current study, intestinal sections of infected untreated animals showed increased IEL numbers, which is in line with a previous report [76]. IELs are part of the immune mechanism to clear *G. lamblia* infection [89]. This rise of IEL numbers could be a response to antigens secreted by the parasite [90]. Unlike immunocompetent animals, nude athymic mice failed to eliminate Giardia infection, suggesting a role for T cells in elimination of the parasite [90]. Both CD4^+^ and CD8^+^ cells contribute to increased IELs during giardiasis [76]. However, increased IELs have been implicated in villus damage and epithelial injury during giardiasis. Scott and colleagues have demonstrated that transplantation of activated CD8^+^ T cell from Giardia-infected animals to normal mice was associated with epithelial tissue damage [76]. Additionally, Ebert demonstrated that *G*. *lamblia* increased proliferation of CD4^+^ T cells and increased secretion of IFN-γ from both intestinal and blood lymphocytes [90]. On the contrary, animals treated with *A. annua* showed lower number of IELs compared to infected untreated animals. This observation could be attributed to the reduction of the parasite load in treated animals.

Goblet cells are essential for protection of the intestinal epithelium from luminal pathogens by secreting mucins which form a protective viscous barrier against invading pathogens [91,92]. In giardiasis, trophozoites colonize the intestine by secreting proteases which disrupt the mucus barrier [93]. Interestingly, Shukla and Sidhu have shown that infection of mice with *G. lamblia* reduced goblet cell numbers, increased excretion of cysts in stool, and decreased trophozoite count in the intestine [94]. Similarly, we show that infection of animals with *G. lamblia* significantly reduced goblet cell number compared to uninfected animals. This reduction in the number of goblet cells could be due to the breakdown of mucin by trophozoites’ proteases [93], and hence could not be detected by Periodic Acid Schiff (PAS) reagent.

In our study, we showed that treating infected hamsters with *A. annua* led to restoration of goblet cell number. This could be attributable to the direct cytotoxic effect of *A. annua* on trophozoites as we mentioned earlier. Therefore, we can also assume that increased goblet cell number and hence mucus production by *A. annua* impaired colonization of the intestine by giardia trophozoites. This assumption is supported by the in vitro experiments of Roskens and Erlandsen who demonstrated that mucin inhibited the attachment of trophozoite to the substratum [95].

A possible limitation of our study should be noted here.We used a high dose of *A. annua* ethanolic extract similar to a number of studies [96,97]. Using a high dose of *A. annua* ethanolic extract ensures sufficient concentration of all active ingredients, particularly polysaccharides, coumarins, saponins, phytosterols, essential oils, polyphenols, and flavonoids [98]. This is supported by the finding that the use of the whole plant is more effective in the treatment of a rodent malaria model, as compared to the use of a comparable dose of pure artemisinin [99].

In conclusion, infection of hamsters with *G. lamblia* lowered NO production, while increasing IL-6, IFN-γ, and TNF-α. Additionally, *G. lamblia* infection reduced villus height, goblet cell number, and muscularis externa thickness, while increasing IEL numbers. Interestingly, treatment with *A. annua* reduced inflammation and pathological damage to the intestine and the therapeutic effect was comparable to that of metronidazole. Treatment of infected animals with *A. annua* extract also reduced small intestinal trophozoite count. Therefore, we believe that *A. annua* extract could be a potential therapeutic option for giardiasis. 

## 5. Conclusions 

The increase in resistance of *G. lamblia* to commonly used anti-giardia agents necessitates the development of novel therapeutics. In this study, we show that *A. annua* extract is effective in controlling giardiasis in a hamster disease model. Furthermore, we demonstrate that the *A. annua* exerts its therapeutic effect by reducing trophozoite count, alleviating inflammation, and reversing pathological damage to the intestinal wall. Therefore, we believe that *A. annua* could be a potential effective therapeutic for the management of *G. lamblia* resistant strains.

## Data Availability

Not applicable.

## List of Abbreviations

*A. annua*	*Artemisia annua*
HPF	High power field
IEL	Intraepithelial Lymphocytes
IL-6	Interlukin-6
IFN-γ	Interferon-γ
iNOS	Inducible nitric oxide synthase
NF-kB	Nuclear factor-kappa B
nNOS	Neuronal nitric oxide synthase
NO	Nitric oxide
PAS	Periodic Acid Schiff reagent
TNF-α	Tumor necrosis factor-α
TNFR1	TNF-α receptor 1
